# Radiation alters PD-L1/NKG2D ligand levels in lung cancer cells and leads to immune escape from NK cell cytotoxicity via IL-6-MEK/Erk signaling pathway

**DOI:** 10.18632/oncotarget.19193

**Published:** 2017-07-12

**Authors:** Ming Jing Shen, Li Jun Xu, Li Yang, Ying Tsai, Peter C. Keng, Yongbing Chen, Soo Ok Lee, Yuhchyau Chen

**Affiliations:** ^1^ Department of Radiation Oncology, University of Rochester School of Medicine and Dentistry, Rochester, NY 14642, USA; ^2^ Department of Cardiothoracic Surgery, The Second Affiliated Hospital of Soochow University, Suzhou, Jiangsu 215004, P.R. China

**Keywords:** PD-L1, NKG2D, NK cytotoxicity, MEK/Erk, radioresistant lung cancer

## Abstract

We investigated whether radiation influences the susceptibility of non-small cell lung cancer (NSCLC) cells to NK cell mediated cytotoxicity. We found radiation treatment increased expression of programmed cell death ligand 1 (PD-L1), but decreased NK group 2, member D (NKG2D) ligand expressions in A549 and H157 NSCLC cells. Both types of changes would have protected tumor cells from the cytotoxic action of NK cells. Consistently, we detected similar alteration in these molecules in radioresistant A549R26-1 and H157R24-1 subline cells. Higher PD-L1 level was also observed in tumors of A549R26-1 cell-derived xenografts than tumors of parental A549 (A549P) cell-derived xenografts. Accordingly, we found radioresistant cells were more resistant to the cytotoxic action of NK cells than parental cells, and such resistance was decreased when neutralizing antibody (Ab) of PD-L1 was added to the radioresistant cell/NK cell co-cultures. In mechanism studies, we found that IL-6-MEK/Erk signaling contributed most significantly to the up-regulation of PD-L1/down-regulation of NKG2D ligands in radioresistant cells. The addition of the MEK/Erk inhibitor increased the susceptibility of A549R26-1 and H157R24-1 cells to NK-cell cytotoxicity while no significant effect was observed in parental cells. Moreover, we detected enhanced NK-cell cytotoxicity to radioresistant cells when PD-L1 Ab and MEK/Erk inhibitor were added together to co-cultures of tumor/NK cells compared to when PD-L1 Ab was used alone. We suggest that combined use of PD-L1 Ab and MEK/Erk inhibitor may offer better therapeutic benefits than PD-L1 Ab alone to treat NSCLC patients who are receiving radiotherapy or who are at the radioresistant stage.

## INTRODUCTION

Primary radiation therapy (RT) is effective in treating non-small cell lung cancer (NSCLC) patients, especially those who are unable to tolerate the rigors of surgery for early stage NSCLC, or those who have locally advanced inoperable NSCLC [1]. Meanwhile, recent clinical trials have demonstrated effectiveness of immunotherapy for advanced NSCLC [2, 3], which is now being tested in combination with radiotherapy [4]. Among immunotherapeutic options, blocking the programmed death cell receptor ligand 1(PD-L1)/PD-1 immune checkpoint has been the major strategy to enhance T cell-mediated immune function [5].

While the PD-L1 (tumor cells)/PD-1 (NK/T cells) axis represents a critical inhibitory immune checkpoint, the natural killer (NK) group 2D (NKG2D) ligand (tumors cells)/NKG2D (NK/T cells) is recognized as a stimulatory axis that promotes immune function. Thus, in addition to the up-regulation of PD-L1, the down-regulation of NKG2D activating ligands such as UL16 binding protein 1 (ULBP1), ULBP2, ULBP3, major histocompatibility complex class I chain-related molecules A and B (MICA, and MICB), is another way for tumor cells to escape from NK cell-mediated cytotoxic action [6]. Therefore, NKG2D ligands have emerged as another important target in immunotherapy [7].

It has been suggested that RT can impact anti-tumor immune responses [8]. Kachikwu *et al.* [9] showed that radiation enhanced regulatory T cell presentation, and Schaue *et al.* [10] reported that fractionated RT helped tumor immunity by increasing reactive T cell numbers. It was also suggested that radiation treatment-induced substantial changes in the tumor microenvironment (TME) and changes in pro-inflammatory cytokines, chemokines, and immunosuppressive T cell subsets, as well as in immune receptors on tumor cells, thereby directing to anti-tumor immune environments [4]. In addition, delivery of localized RT to tumors often leads to systemic responses at distant sites, a phenomenon known as the abscopal effect, which has been attributed to the induction and enhancement of the endogenous anti-tumor innate and adaptive immune response [11]. Deng *et al.* showed that irradiation and anti-PD-L1 treatment synergistically promoted antitumor immunity in mice [12]. The synergy of RT and PD-1 blockade in Kras-mutant lung cancer has also been reported [13].

However, contradictory to this concept that radiation may help immune reaction, we recently found that repetitive irradiation increased PD-L1 level while decreased NKG2D ligand levels in NSCLC cells. As high levels of PD-L1 and low levels of NKG2D ligands in tumor cells would have been involved in immune escape process, we studied whether the radiation-induced up-regulation of PD-L1/down-regulation of NKG2D ligands might induce lower susceptibility of lung tumor cells to cytotoxic actions of NK cells. As such a radiation-induced effect may be reversible, we developed radioresistant NSCLC sub-line cells that did exhibit constitutive expression of PD-L1 and lower NKG2D ligand levels. We used these cells in studying the association of radiation effects with the development of resistance to cytotoxic actions of NK cells.

We have focused on the immune escape of radioresistant cells from NK-cell cytotoxicity as interests in NK-cell mediated cytotoxicity to control tumor development and progression is increasing. It has also been suggested that cancers develop mechanisms to escape NK cell attack or induce defective NK cells [14]. Decreased numbers of NK cells in cancer patients also indicate the importance of NK cells in combating early stage tumor development [15, 16]. The evidence showing effects of anti-PD-L1/PD-1 strategy in increasing NK cell-mediated action is emerging. For example, the anti-PD-L1/PD-1 effects in enhancing NK cell function in multiple myeloma was demonstrated [17] and several *in vitro* results were reported [18, 19]. In this study, we aimed to develop a therapeutic strategy for lung cancer patients who will receive RT or are at the radioresistant stage by targeting the signaling pathway that is responsible for the radiation-induced PD-L1 increase and NKG2D ligands decrease.

Thought to be involved in the modulation of the radiation-induced PD-L1 increase and NKG2D ligands decrease in lung cancer cells after radiation, we studied the implication of IL-6 signaling based on our several previous findings. In previous investigations, we observed significant increase of IL-6 level in lung cancer cells after radiation treatment [20], which was matched with the earlier report showing the radiation-induced IL-6 level increase in lung cancer cells [21]. We also found that IL-6 signaling was involved in rendering radioresistance of lung cancer cells by promoting DNA repair after irradiation [22]. These findings were also consistent with reports showing the implication of IL-6 signaling in radioresistance development in several other types of cancers [23–26]. Nevertheless, whether a link exists between the IL-6 signaling-triggered radioresistance development and the resistance induction to NK cell cytotoxicity in lung cancer is not known. In this study, we aimed to elucidate the association of these two processes.

## RESULTS

### Radiation increased PD-L1, while reduced NKG2D ligand levels in lung tumor cells

**Figure 1:**
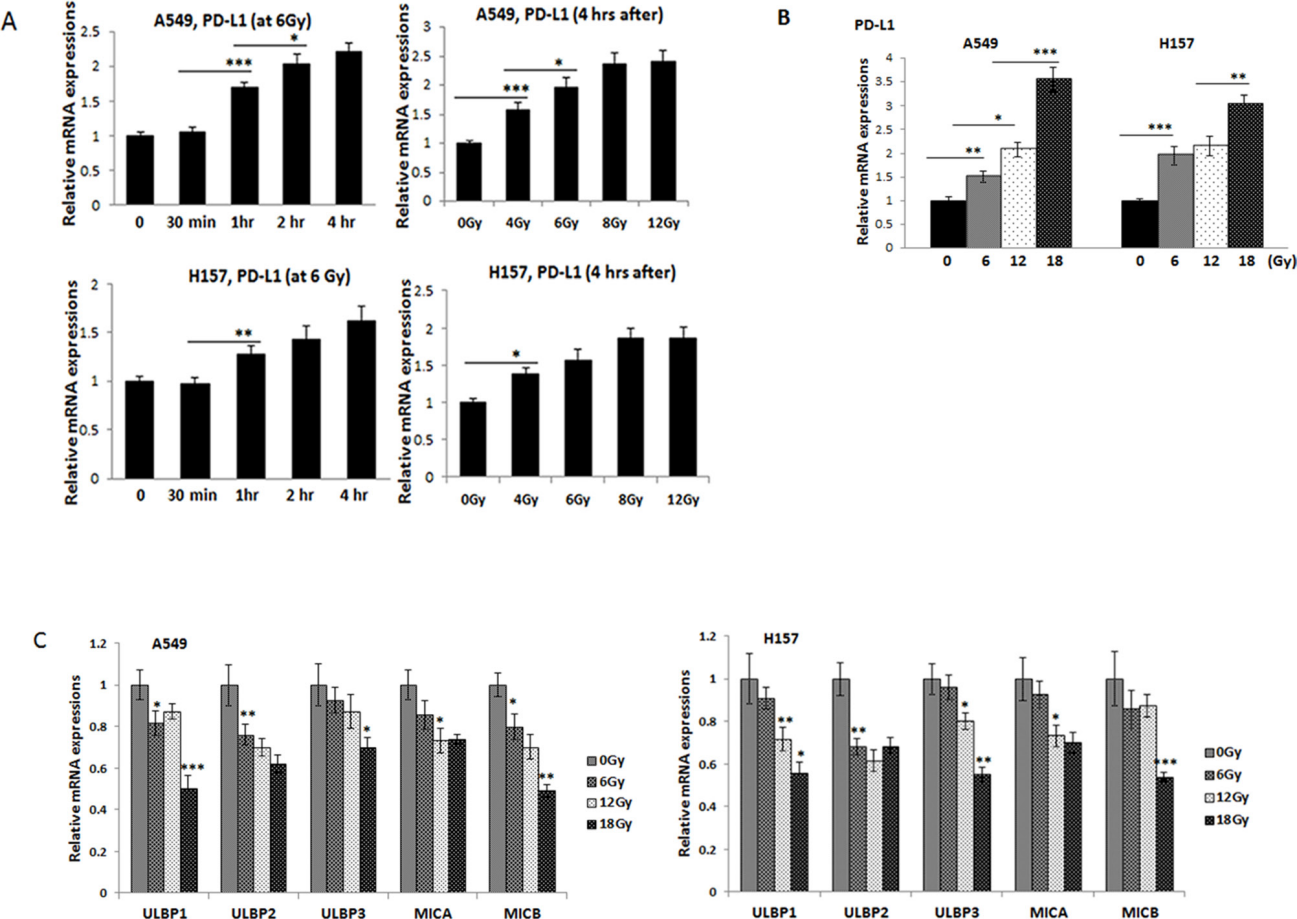
Investigations on the PD-L1 and NKG2D ligand levels in lung cancer cells after radiation treatment (**A**) qPCR analyses of PD-L1 mRNA expressions in A549 and H157 cells after radiation; at various time points (upper panel) and various radiation doses (lower panel). Total mRNAs were extracted from non-radiated and radiated cells, cDNA converted, and expressions of PD-L1 were analyzed. (**B**) qPCR analyses of PD-L1 levels after treated radiation with 6, 12, and 18 Gy (accumulated) radiation. A549 and H157 cells were treated with radiation (6 Gy) and the survived cells after radiation were further treated with more radiation (6 Gy, one week interval to a total of 12 Gy or 18 Gy), qPCR analyses were performed similarly. (**C**) qPCR analyses of five NKG2D ligands, ULBP1, ULBP2, ULBP3, MICA, and MICB in irradiated and non-irradiated A549 and H157 cells. *p < 0.05, **p < 0.01, ***p < 0.001

To investigate whether radiation influences the PD-L1 level in lung tumor cells, we analyzed the PD-L1 level in irradiated and non-irradiated A549 and H157 cells. We found that radiation treatment increased PD-L1 level in these cells in time-dependent (Figure 1A, left panels) and dose-dependent (Figure 1A, right panels) manners. When surviving cells were further treated with radiation (6 Gy × 1, 6 Gy × 2, 6 Gy × 3, and 6 Gy × 4), we found a more significant PD-L1 increase with accumulated radiation treatments (Figure 1B).

### Radiation reduced NKG2D ligands levels in lung tumor cells

We also investigated whether radiation influences the NKG2D ligands levels, which are known to promote the interaction between tumor cells and NK cells [6]. We analyzed expressions of the five NKG2D ligands, ULBP1, ULBP2, ULBP3, MICA, and MICB (Figure 1C). We found levels of these two ligands decreased in irradiated cells compared to non-irradiated cells. These results suggest that radiation altered not only the PD-L1 level, but also the NKG2D ligands levels in NSCLC cells.

### Development of radioresistant NSCLC cells

**Figure 2:**
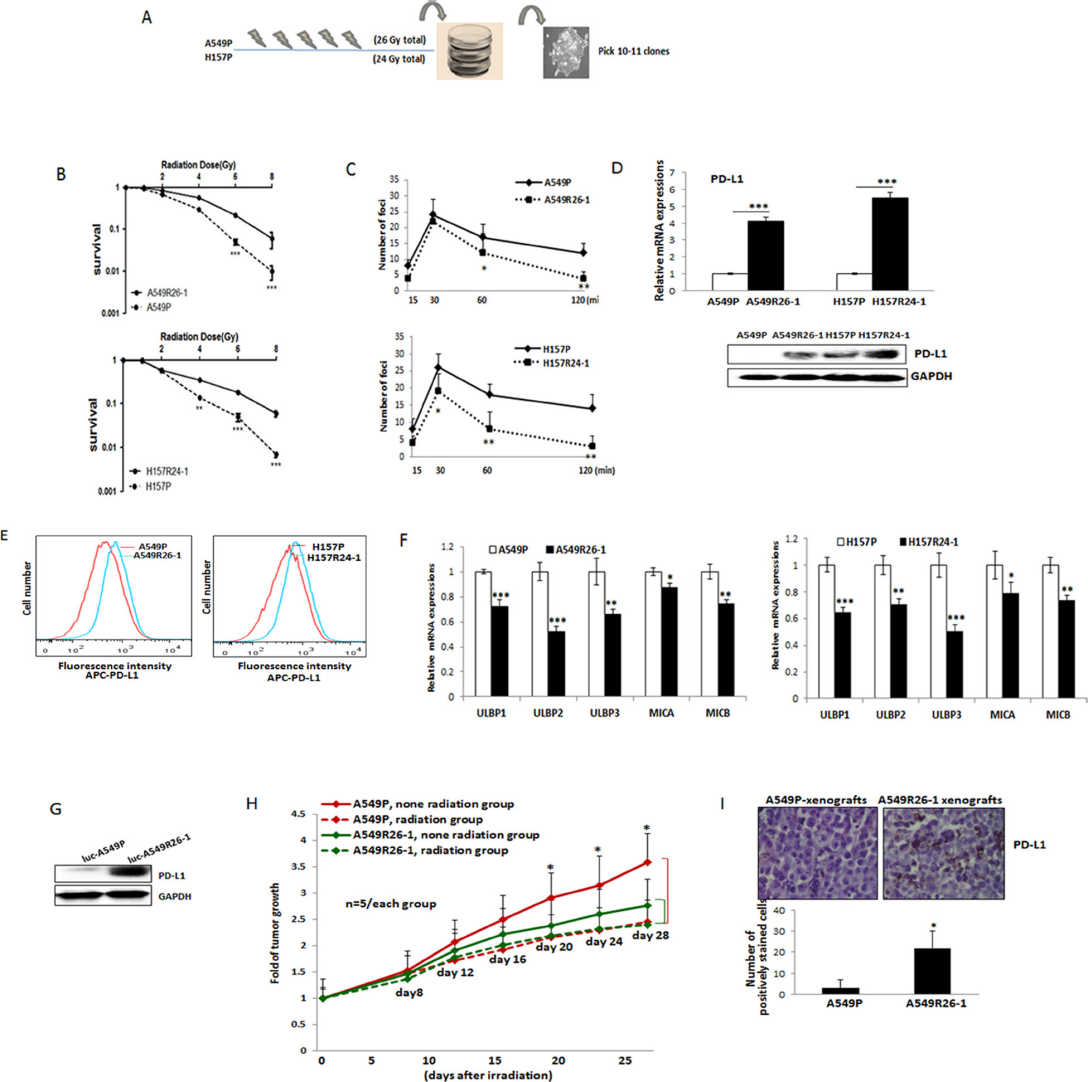
PD-L1/NKG2D ligand levels in radioresistant NSCLC sub-line cells and radioresistant cell-derived tumors (compared to parental cells and parental cell-derived tumors) (**A**) An illustration of radioresistant sub-line development procedure. (**B**) Clonogenic assay showing radioresistance of A549R26-1 and H157R24-1 sublines vs. respective parental cells. 100 to 1,000 cells (A549P/A549R26-1 and H157P/H157R24-1) were plated and cell survivals after different doses of radiation treatment were analyzed in clonogenic assay. (**C**) γH2AX IF staining. The numbers of γH2AX foci (A549P/A549R26-1 and H157P/H157R24-1) at different time points after radiation (6 Gy) were analyzed. (**D**) PD-L1 expression in parental and radioresistant cells (upper panel, qPCR data; lower panel, Western blot data). (**E**) Flow cytometry results analyzing surface PD-L1 levels in parental (red) and radioresistant (blue) cells. (**F**) Expression of NKG2D ligands (mRNA) in parental vs. radioresistant cells. (**G**) Western blot analysis results showing the PD-L1 levels in injected luc-549R26-1 and luc-A549P cells. (**H**) Mice studies testing radioresistance. Tumor regression differences at each time point in A549P-xenografts vs. A549R26-1 xenografts, with or without radiation treatments (5 Gy × 5 days) is shown. Y-axis represents fold of tumor growth based on luminescence measurement. (**I**) IHC staining of PD-L1 in tumor tissues obtained from A549P cells-derived and A549R26-1 cells-derived xenografts. *p < 0.05, **p < 0.01, ***p < 0.001

The observed radiation effects on altering PD-L1/NKG2D levels may be reversible. To investigate radiation effects on PD-L1/NKG2D ligand expressions in lung cancer cells in a better model, we developed radioresistant sublines of A549 and H157 cells. To obtain these sub-lines, parental A549P and H157P cells were treated with 2-6 Gy radiation every week for 4-5 weeks (cumulative total 26 Gy of radiation was applied to A549P cells and 24 Gy to H157 cells). By seeding cells at low density, we selected 10-11 sub-colonies (selection procedure described in Figure 2A). Cell extracts of sub-lines were obtained and the expression of PD-L1 was investigated. As shown in Supplementary Figure 1A, almost all sub-lines showed higher levels of PD-L1 than parental cells. Three sub-lines showing high PD-L1 level were selected, and whether these cells were radioresistant was tested in clonogenic assays. From these analyses, we finally selected one subline of each cell line, A549R26-1 and H157R24-1 that exhibited the most significant resistance to radiation. Figure 2B is the clonogenic assay result of A549R26-1 and H157R24-1 cells vs. respective parental cells (Supplementary Figure 1B shows the plate images). The γH2AX IF staining result (Figure 2C and Supplementary Figure 1C) showing higher DNA damage recovery of A549R26-1 and H157R24-1 cells upon radiation than parental cells supported the clonogenic assay result. To test whether high PD-L1 expression level in those cells is associated with radioresistance, we selected one A549 sub-line cell that showed low PD-L1 level and performed radiation clonogenic assay. We found the radiation sensitivity of these cells was like that of parental cells (Supplementary Figure 2), suggesting that the level of PD-L1 in subline cells with high expressions may be associated with radioresistance.

### Up-regulated PD-L1, but reduced NKG2D ligands levels in radioresistant lung cancer cells compared to parental cells

We investigated whether PD-L1 level is higher in radioresistant cells than parental cells. Without radiation treatment, we detected constitutively expressed PD-L1 in A549R26-1 and H157R24-1 cells compared to parental cells (Figure 2D, left panel, mRNA level; right panel, protein level). Higher level of cell surface PD-L1 expression was also observed in radioresistant cells than in parental cells as shown in flow cytometric analysis (Figure 2E). We also tested the NKG2D ligand levels in radioresistant cells. We found levels of the 5 NKG2D activating ligands, ULBP1, ULBP2, ULBP3, MICA, and MICB were all significantly down-regulated in A549R26-1 and H157R24-1 cells compared to parental cells (Figure 2F).

### Development of mouse models bearing radioresistant tumors

To test whether radioresistant tumors express higher levels of PD-L1 than radiation sensitive tumors *in vivo*, orthotopic xenograft mouse models were developed in nude mice by injection of luciferase tagged A549P and A549R26-1 cells, as previously published reports also have shown the development of resistant tumors by injecting radioresistant sub-cell line cells [27, 28]. The PD-L1 levels in luciferase tagged cells are shown in Figure 2G. The tumor bearing mice were similarly irradiated (5 Gy/day, 5 days) when luminescence reached to 5 × 10^5^ to 1 × 10^6^ radiance and tumor growth was monitored for 3–4 weeks. We observed significant tumor regression in A549P cells-derived xenografts, but not in the A549R26-1 cells-derived one (Figure 2H). As an example, the IVIS image of one mouse in each group is shown in Supplementary Figure 3, suggesting that injection of radioresistant cells successfully developed radioresistant tumors *in vivo*. When we examined PD-L1 levels in radioresistant tumors (A549R26-1 cells-derived) and radiation-sensitive (A549P cells-derived) tumors, we detected higher PD-L1 expression in tumor tissues of A549R26-1 cells-derived xenografts than in tumors of A549P-xenografts (Figure 2I).

### Radioresistant cells were resistant to NK cell cytotoxicity

**Figure 3:**
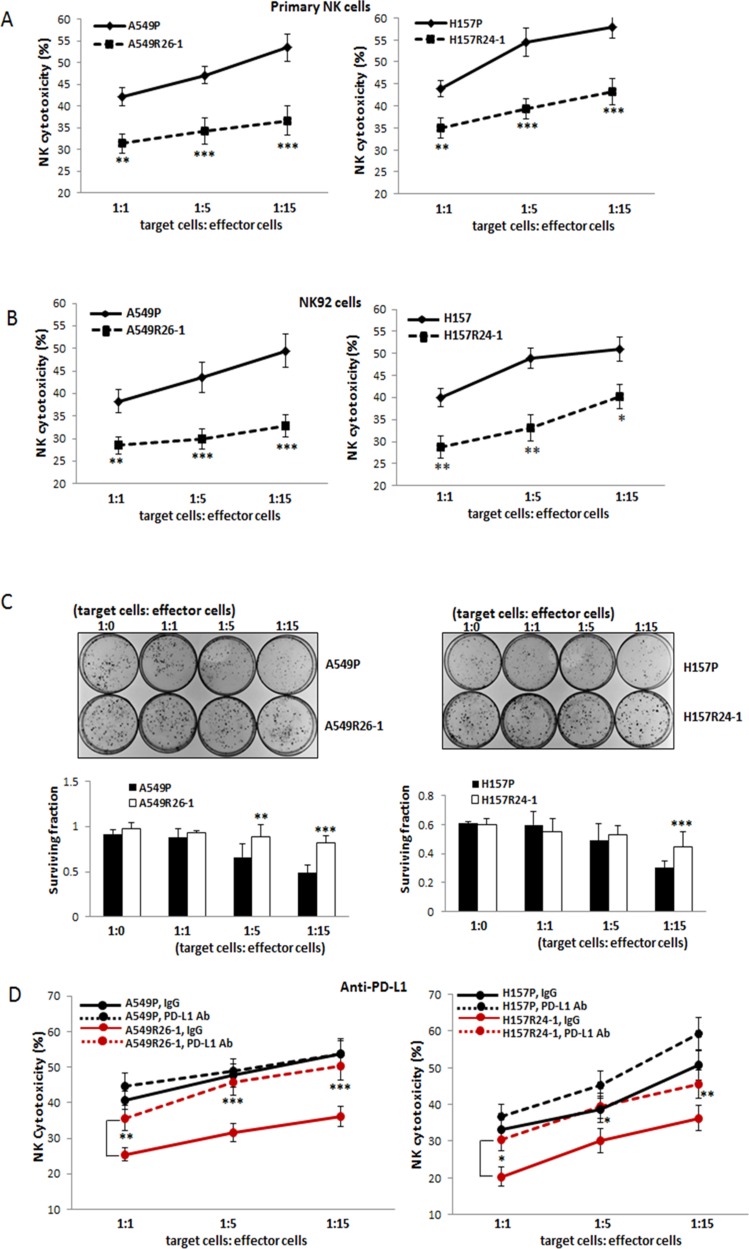
Tests of susceptibilities of radioresistant cells vs. parental cells to NK cell cytotoxicity (**A, B**) NK-cell cytotoxicity tests using primary NK cells (A) and NK92 cells (B). The susceptibilities of A549P/A549R26-1 and H157P/H157R24-1 cells to NK cell cytotoxicity were tested in LDH release-based cytotoxicity test kit. The experimental release was corrected by subtraction of the spontaneous release of effector cells at corresponding dilutions. Left panels show data of A549P/A549R26-1 cell set and right panels show data of H157P/H157R24-1 cell set. (**C**) Colony formation assay. The surviving A549P/A549R26-1 and H157P/H157R24-1 cells after NK cell incubation (4 hours) were grown. The colonies were stained with Crystal Violet and the colony numbers were counted under microscopy. (**D**) NK cell cytotoxicity test after adding PD-L1 Ab (matched IgG as control) to tumor/NK cells co-culture. *p < 0.05, **p < 0.01, ***p < 0.001

As we observed higher PD-L1 and lower NKG2D ligands in radioresistant cells, we hypothesized that these cells are more resistant to NK cell cytotoxicity than parental cells and tested this by performing NK cytotoxicity tests. We used two NK cell sources, an established cell line NK92 that has been known to exhibit high NK cytotoxicity and widely used in *in vitro* and in mouse studies [29, 30], and primary NK cells that were isolated from the peripheral blood mononuclear cells (PBMCs) of healthy donors via magnetic beads isolation method. After isolation of primary NK cells, the purity of CD56+CD3- NK cells were confirmed (higher than 90%) by flow cytometric analysis (data not shown). For monitoring NK-cell mediated cytotoxicity, two different assays, the lactate dehydrogenase (LDH) release-based NK cytotoxicity test [31–34] and the colony formation assay [35] were used. In LDH-based assay, we found radioresistant cells were more resistant to the cytotoxic action of NK cells than parental cells (Figure 3A, data with primary NK cells; 3B, data with NK92 cells). In colony formation assays, the colonies developed from surviving cells after co-culture with NK cells were visualized. We observed higher colony numbers of A549R26-1 and H157R24-1 cells than in parental cells after co-culture with NK92 cells, suggesting lower susceptibility of radioresistant cells than parental cells to NK cell cytotoxicity (Figure 3C). The results of both assays suggest that radioresistant lung cancer cells were more resistant to NK-cell mediated cytotoxic action than parental cells.

### Increased NK cell cytotoxicity to radioresistant cells when PD-L1 Ab was added to co-culture

NK-cell cytotoxicity to A549R26-1 vs. A549P and H157R24-1 vs. H157P cells were tested after adding the neutralizing Ab of PD-L1 (or control IgG) into the tumor cell/NK cell co-culture. As shown in Figure 3D, the PD-L1 Ab addition significantly increased the susceptibilities of A549R26-1 and H157R24-1 cells to the cytotoxic action of NK cells, but the susceptibilities of parental cells A549P and H157P were not significantly influenced. Such result suggests that disrupting the PD-L1/PD-1 interaction was more effective in enhancing the susceptibilities of radioresistant cells to NK cells cytotoxicity than parental cells.

### The radiation-induced, increased IL-6 signaling was responsible for the up-regulation of PD-L1/ down-regulation of NKG2D ligands in radioresistant cells

We then explored signaling pathways that may be responsible for the up-regulation of PD-L1 and down-regulation of NKG2D ligands in radioresistant cells. We investigated the implication of IL-6 signaling in this regulation as our laboratory found that IL-6 level significantly increases upon radiation [20] and such an increase was associated with radioresistance development [22].

**Figure 4:**
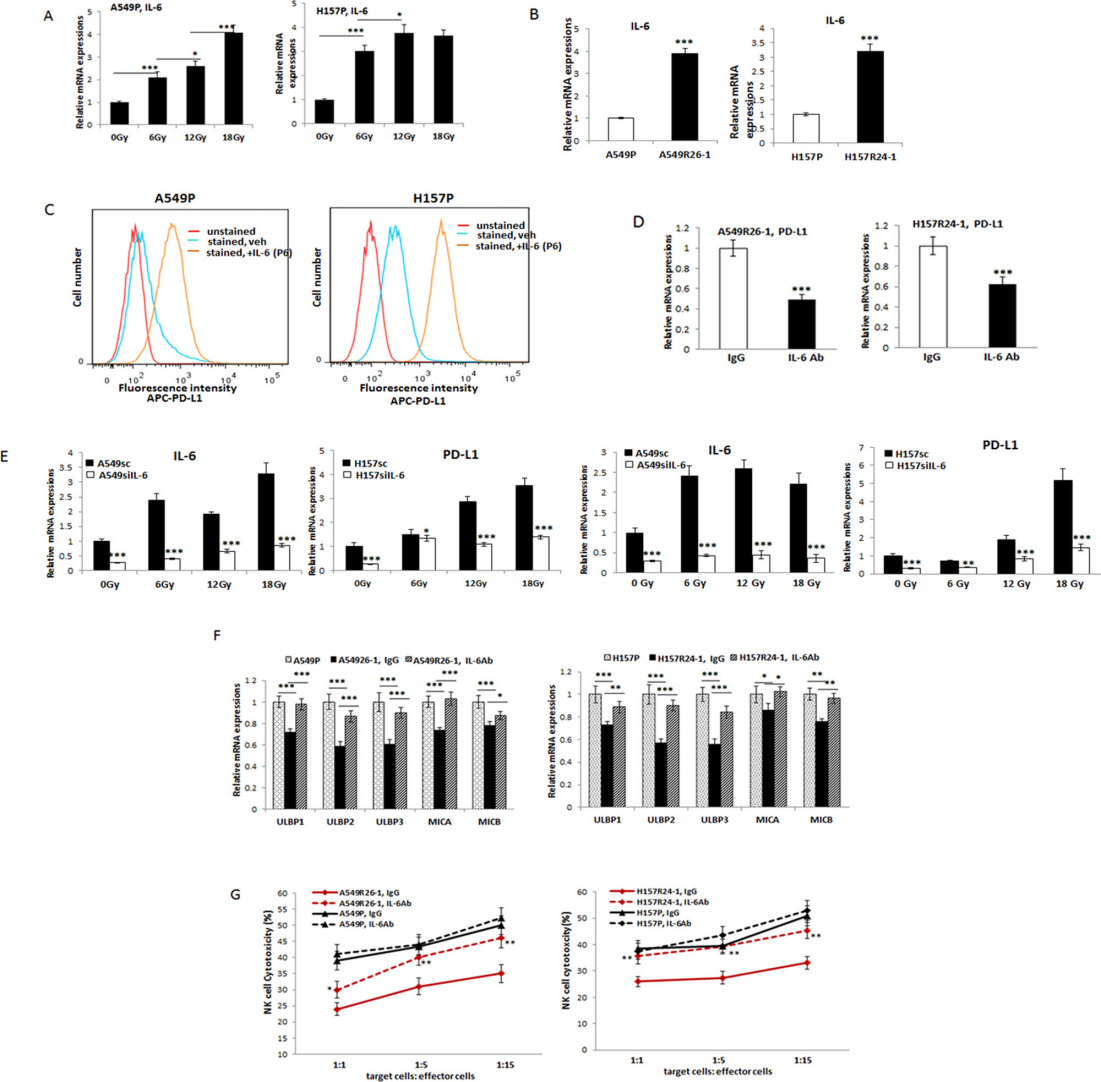
Investigations on the implication of IL-6 signaling in altering PD-L1/NKG2D ligand levels in lung cancer cells and its influence on NK cell cytotoxicity to tumor cells (**A**) qPCR analyses of IL-6 levels in A549 and H157 cells after radiation. (**B**) IL-6 levels (mRNA) in parental and radioresistant cells. (**C**) Flow cytometric analysis of surface PD-L1 levels on A549 and H157 cells after treating with IL-6 (P6; passage 6 in the presence of IL-6, 10 ng/ml). (**D**) PD-L1 level in A549R26-1 and H157R24-1 cells after treating cells with IL-6 Ab (IgG as control). (**E**) qPCR analyses of PD-L1 in A549siIL-6/sc and H157siIL-6/sc cell sets after radiation IL-6 knocked down (siIL-6) and scramble (sc) control cells were treated with radiation (6, 12, and 18 Gy total) and PD-L1 levels were analyzed by qPCR analyses. (**F**) NKG2D ligand levels in A549R26-1 and H157R24-1 cells after treating cells with IL-6 Ab (IgG as control). (**G**) NK cell cytotoxicity test to A549R26-1 and H157R24-1 cells after adding IL-6 Ab to the co-culture of tumor/NK cells. Primary NK cells were used in this assay. *p < 0.05, **p < 0.01, ***p < 0.001.

The IL-6 level increase in A549P and H157P cells after radiation treatment is shown in Figure 4A. When we tested the IL-6 level in radioresistant cells vs. parental cells, we also found increased IL-6 levels in radioresistant A549R26-1 and H157R24-1 cells than parental cells (Figure 4B).

To investigate possible correlation of IL-6 and PD-L1 level increases after radiation, we treated parental A549P and H157P cells with IL-6 and tested whether the surface PD-L1 level increases in these IL-6 treated cells. As shown in Figure 4C, the PD-L1 level increase in A549P and H157P cells was observed upon continuous treatment of IL-6 (the data of passage 6 with IL-6 treatment is shown). The correlation of IL-6 and PD-L1 level increases after radiation was also observed in experiments with radioresistant cells. When A549R26-1 and H157R24-1 cells were treated with the neutralizing antibody (Ab) of IL-6 (vs. control IgG), we found the PD-L1 level was significantly decreased (Figure 4D). The implication of IL-6 signaling in radiation-induced PD-L1 increase was further shown in experiments using the IL-6 knocked down (siIL-6) and scramble (sc) control (A549siIL-6/sc and H157siIL-6/sc) cell sets. We found more significant IL-6 expression differences in IL-6 knocked-down vs. control cells after radiation and the up-regulation profiles of IL-6 and PD-L1 in siIL-6 and sc cells upon radiation were also similar (Figure 4E).

**Figure 5:**
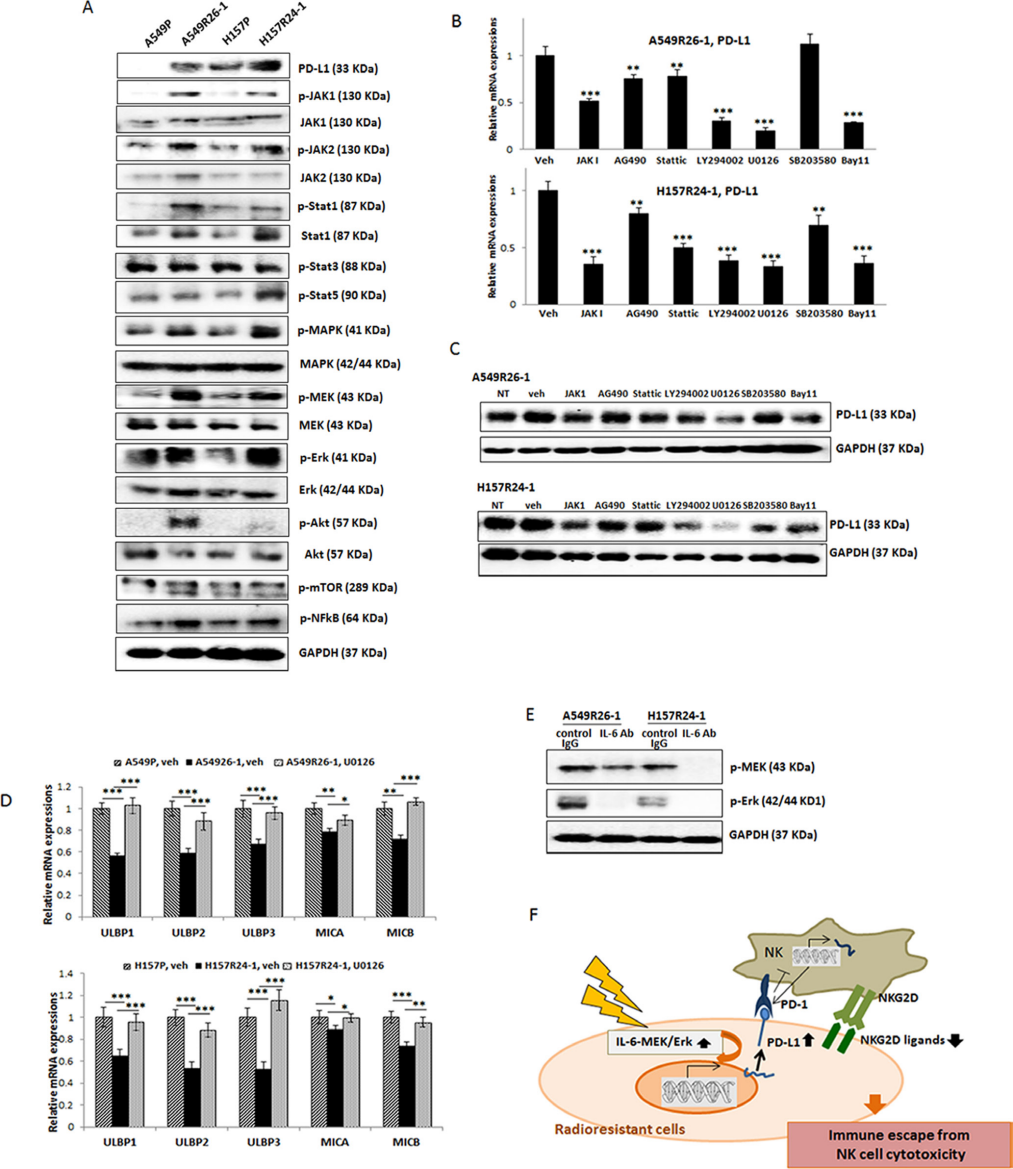
Revealing signaling pathways that are responsible for the PD-L1 level increase/NKG2D ligand level decrease in A549R26-1 and H157R24-1 cells (**A**) Western blot analyses showing expression/activation of several signaling molecules in parental and radioresistant cells. (**B**) Western blot analyses showing inhibition of each pathway in A549R26-1 cells upon inhibitor treatment. (**C** and **D**) qPCR analyses (C) and Western blot analyses (D) of PD-L1 levels in A549R26-1 and H157R24-1 cells upon treatment with inhibitors of indicated signaling pathways. (**E**) Western blot analysis showing p-MEK/p-Erk levels after adding IL-6 Ab (control IgG as control) in radioresistant cells. (**F**) A figure describing the mechanism leading to the resistance to NK cell cytotoxicity. *p < 0.05, **p < 0.01, ***p < 0.001.

We also found that the NKG2D ligands levels (Figure 4F) were recovered when radioresistant cells were incubated with the IL-6 Ab, suggesting that IL-6 signaling may also alter the NKG2D ligands level upon radiation. We then tested the susceptibilities of A549R26-1 and H157R24-1 cells to NK cell cytotoxicity after adding IL-6 Ab. We found the susceptibilities were significantly enhanced after IL-6 Ab treatment (Figure 4G). All these results in Figure 5 indicate that IL-6 signaling increase was critical in PD-L1/NKG2D ligand level alteration in irradiated lung cancer cells, and thus could control the NK cell cytotoxicity to tumor cells.

### Identification of IL-6 downstream signaling pathway that mediates constitutive expression of PD-L1/down-regulation of NKG2D ligands in radioresistant cells

We then searched IL-6 downstream signaling pathways that may be responsible for the up-regulation of PD-L1/down-regulation of NKG2D ligands in radioresistant cells. We first analyzed expression/activation of IL-6 downstream signaling molecules in parental vs. radioresistant cells which have been previously reported to be involved PD-L1 up-regulation. These included: JAK1/2 [36, 37], Stat 1 [36], Stat3 [38, 39], Stat5 [40], NFκB [41], Mitogen-*activated* protein kinase/ERK kinase (*MEK*), extracellular-signal-regulated kinase (*ERK*) [42], PI3K/Akt [43, 44], and MAPK [45] pathways. We found several signaling pathways, including JAKs, Stats, MAPK, and Erk were activated in radioresistant cells compared to parental cells (Figure 5A). Using inhibitors of each signaling pathway, we then tested the blocking of the signaling pathway(s) that could reduce the constitutive expression of PD-L1 in radioresistant cells. We detected inhibitors of JAK, MAPK, MEK/Erk, and Stat signaling that reduced PD-L1 levels, but the most significant effect was observed with the MEK/Erk inhibitor (U0126) treatment (Figure 5B, qPCR data; 5C, Western blot data).

Further, we investigated whether the MEK/Erk inhibitor also influenced the expression of the NKG2D ligands in radioresistant cells. We found that the inhibitor treatment recovered NKG2D ligands levels (Figure 5D), indicating that inhibiting the MEK/Erk signaling was effective not only in inhibiting the constitutive expression of PD-L1, but also in recovering the expression of the reduced NKG2D ligands. The IL-6-regulation of MEK/Erk signaling was confirmed by showing reduced p-MEK and p-Erk levels in radioresistant cells when these cells were incubated with IL-6 Ab (Figure 5E).

### MEK/Erk signaling inhibitor increased the NK cell cytotoxicity to radioresistant cells and the combined use of this inhibitor enhanced the PD-L1 effect

**Figure 6:**
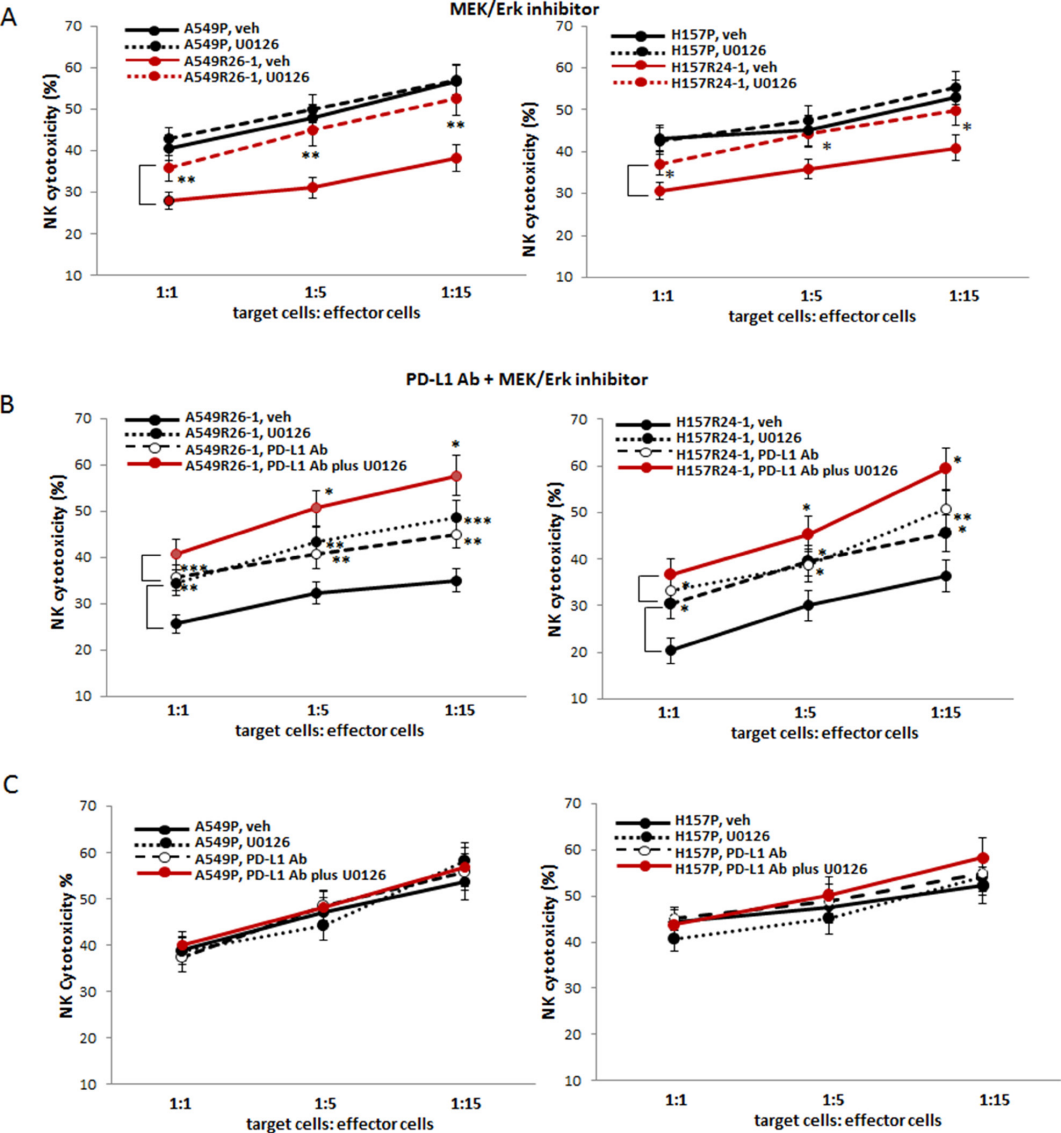
Testing susceptibilities of radioresistant lung cancer cells to NK cell cytotoxicity after adding the inhibitor of MEK/Erk signaling pathway (U0126) (**A**) NK cell cytotoxicity tests to A549R26-1 and H157R24-1 cells after adding U0126 (vehicle as control) to tumor/NK cell co-culture. (**B** and **C**) NK cell cytotoxicity tests to A549R26-1 and H157R24-1 cells after adding both PD-L1 Ab and U0126 to tumor cell/NK cell co-cultures (B represents data with A549R26-1/H157R24-1 cells and C shows data with parental cells). *p < 0.05, **p < 0.01, ***p < 0.001.

The effect of MEK/Erk inhibitor (U0126) on increasing NK cell cytotoxicity to radioresistant cells was tested. We found that the MEK/Erk inhibitor treatment significantly increased NK cell cytotoxicity to A549R26-1 and H157R24-1 cells (Figure 6A) similarly to the effect observed with the PD-L1 Ab (Figure 3G), but such effect was not detected in experiments with parental cells (Figure 6A).

Finally, we tested the effect of combined use of the MEK/Erk inhibitor and the PD-L1 Ab. We discovered that the combined use of MEK/Erk inhibitor (U0126) with PD-L1 Ab demonstrated significantly enhanced effects compared to the effects of PD-L1 Ab alone or MEK/Erk inhibitor alone (Figure 6B, A549R26-1/H157R24-1 cell data; 6C, parental cell data). These results provided the potential of combined use of the MEK inhibitor and PD-L1Ab for future clinical applications to prevent the development of immune escape from NK cell mediated cytotoxicity during RT or to target lung cancer patients at the radioresistant stage.

## DISCUSSION

While most studies so far have been concentrated on T-cell mediated immune function, our study has focused on NK-cell mediated cytotoxicity. Recent immunotherapy clinical trials have extensively improved the T cell mediated immunity against lung tumors [2, 3], though the outcome is still not satisfactory concerning lung cancer treatment. This may be due to very low and heterogeneous antigenicity of lung cancer cells [46], resulting in deficiencies in antigen processing and presentation on T cells. Release of immunomodulatory cytokines in tumor microenvironment to inhibit T-cell activation is also considered as a factor contributing to therapy failure [47]. Since NK cells play a pivotal role in exerting antigen-independent, innate immune responses, NK-cell targeted immunotherapy may be a better option. The strategy of ex-vivo expanded NK cells injection has also been considered for achieving anti-tumor effects since donor NK cells do not attack non-hematopoietic tissues [48]. However, as Whiteside and Herberman [49] earlier indicated, the role of NK cells in cancer has generally been underestimated.

In this study, we discovered that radioresistant cells were resistant to cytotoxic actions of NK cells by up-regulating PD-L1 while down-regulating NKG2D ligands. Our *in vitro* results clearly demonstrated that: (i) repetitive radiation treatment increased the PD-L1 level while decreasing NKG2D ligand levels in lung cancer cells, (ii) higher expression of PD-L1 in radioresistant cells than in parental cells (*in vitro*) and in radioresistant tumors than in radiation-sensitive tumors (*in vivo*), and (iii) radioresistant cells showed resistance to cytotoxic actions of NK cells.

Such discoveries are challenging since this concept is contrary to the previous reports suggesting that RT increased T cell proliferation and antigen presentation, thereby increasing T cell mediated immunity. However, our results were similar to recent reports showing PD-L1 increase in bladder cancer cells after radiation treatment [50]. Parikh *et al.* [51] also reported that chemoradiotherapy induced PD-L1 and antagonized immunity in HPV-related oropharyngeal cancer. As suggested by Barker *et al.* [52], radiation effects on TME was a complex reaction, so it might be hard to conclude that radiation effect was immunostimulatory or immunosuppressive. Our new discoveries led us to hypothesize that some targeted therapeutic strategies of blocking the radiation effects on inhibiting the NK cells action might be beneficial when RT or combined RT and immunotherapy is applied to lung cancer patients.

To date, blocking the PD-L1/PD-1 immune checkpoint is considered the most efficient immunotherapy [5, 53]. In our study, we also observed effects of using PD-L1 Ab in increasing NK cell cytotoxicity to radioresistant cells. Currently, the use of PD-L1 Ab in clinical trials of treating several types of cancer has been reported. For example, Nivolumab (PD-L1 Ab) was used for the treatment of metastatic lung cancer on or after docetaxel used-chemotherapy and showed antitumor immunity [54, 55]. Based on our data, radioresistant cells express higher PD-L1 while lower NKG2D ligands than parental cells. We expected that adding a strategy of blocking signaling pathways responsible for radiation-induced up-regulation of PD-L1/down-regulation of NKG2D ligands would exhibit better efficacy than using the PD-L1 Ab alone. We discovered that the MEK/Erk signaling was the critical pathway and we indeed observed significantly enhanced effects when inhibitor of this pathway was applied together with the PD-L1 Ab.

In mechanism studies, we found that IL-6 signaling is critical in up-regulation of PD-L1/down-regulation of NKG2D ligands after radiation. Radiation-induced IL-6 level increase has also been shown in our previous studies studying the IL-6 effect on promoting macrophage infiltration into tumor sites after radiation [20]. A recent report showing that IL-6 inhibition of radiation -induced apoptosis in pancreatic cancer cells [4] also supports our finding.

Directly targeting IL-6 signaling may also be an effective strategy. Recently, effects of the combination therapy using IL-6 Ab plus PD-L1 Ab in reducing pancreatic tumor size have been reported [56]. However, we speculate that the use of anti-IL-6 agents may result in complicated and unforeseen untoward outcomes in therapeutics considering the complexity of the physiological activities of IL-6 in producing both pro- and anti-inflammatory effects in the immune system [57]. Thus, we believe the use of MEK/Erk inhibitor may be a better clinical option than applying IL-6 Ab.

In conclusion, our data support applying the MEK/Erk inhibitor combined with PD-L1 Ab to treat lung cancer patients who are receiving RT or who are at the radioresistant stage. In future preclinical and translational studies, the use of MEK inhibitor is recommended than the Erk inhibitor since clinical trials on MEK inhibitor has shown promising results, while the use of the ERK inhibitors showed high toxicity and is still in clinical developmental stage [58].

## MATERIALS AND METHODS

### Cell culture

A549 and H157 cell lines were purchased from the American Type Culture Collection (ATCC, Manassas, VA) and cultured in RPMI 1640 containing 10% FBS. All cells were maintained in a humidified 5% CO_2_ environment at 37°C. NK92 cell line was also purchased from ATCC (Manassas, VA) and cultured in α-MEM media containing sodium bicarbonate (Sigma, M4655), IL-2 (100 units/ml) (Peprotech, 200-02), inositol (0.2 mM), 2-mercaptoethanol (0.1 mM), folic acid (0.02 mM), 12.5 % horse serum (Sigma) and 12.5% FBS (Hyclone). For inhibitor studies, JAK inhibitor 1 (5 μM) (Calbiochem, CAS457081-03-7), AG490 (5 μM) (Sigma, T3404), Stattic (10 μM) (Calbiochem, CAS19983-44-9), LY294002 (5 μM) (Sigma, 440202), U0126 (10 μM) (Cell Signaling, 9903), SB203580 (10 μM) (Sigma, 559387), Bay11-7082 (5 μM) (Sigma, B5556) that inhibit JAK, JAK/Stat3, Stat3, PI3K/Akt, MEK/Erk, MAPK, and NFκB pathways, respectively, were added into the co-culture of tumor cells/NK cells.

### Development of radioresistant cell lines

Parental A549P and H157P cells were treated with 2–6 Gy radiation treatment every week for 4-5 weeks. Every week, after the recovery of survived cells, further radiation treatment was applied. After receiving a cumulative total of 26 Gy (for A549P cells) and 24 Gy (for H157 cells), the surviving cells were plated at low density. Ten to eleven colonies were picked, expanded, and the radioresistance of each sub-line was tested in clonogenic assays and γH2AX IF staining.

### Clonogenic assay

Cells were exposed to different doses (0, 1, 2, 4, 6, and 8 Gy) of radiation using a Cs-137 source with a dose rate of 180–205 cGy/min. After treatment, clonogenic assay was performed as previously described [59]. Cells were seeded in culture dishes with appropriate dilutions to form colonies after 7–9 days of incubation. Colonies were fixed with methanol, stained with crystal violet (0.5% w/v), and counted under the microscope. Colonies consisting of at least 50 cells were counted and the surviving fraction was calculated after adjusting for the plating efficiency.

### γH2AX immunofluorescence (IF) staining

Cells were exposed to 6 Gy of radiation and stained with γH2AX Ab at different time points (0, 15 min, 30 min, 60 min, 120 min). After incubating with secondary Ab Alexa fluor^®^ 488 tagged goat anti-rabbit) (Life Technologies, A11034), images were obtained under fluorescence microscope.

### Isolation of primary NK cells

Primary NK cells were purified from peripheral blood mononuclear cells (PBMCs) of healthy donors using the NK cells isolation kit (Miltenyi Biotec, 130-092-657) according to manufacturer's protocol. After isolation, the isolated cells were maintained in IL-2 containing NK cell media. The purity of isolated cells (CD56+CD3-) was confirmed in flow cytometric analysis by staining with anti-CD56-PE (e-Bioscience, 12-0267-41) and anti-CD3-Cy7 (BioLegend, 300429) antibodies.

### NK cytotoxicity tests (LDH release-based)

NK cells cytotoxicity against tumor cells (A549P/A549R26-1 and H157P/H157R24-1) was analyzed using a lactate dehydrogenase (LDH) release assay. Cells (2,500 to 5,000) were plated and on the next day NK cells were added at various ratios (1:1, 1:5, and 1:15, target cells: effector cells) (all samples in triplicates). After 4 hours of co-culture, an aliquot of 50 μl media was used in LDH cytotoxic assay using the LDH cytotoxic assay kit (Thermo Scientific, 88954). The experimental release was corrected by subtraction of the spontaneous release of effector cells at corresponding dilutions. %Cytotoxicity = (Experimental value − Effector Cell Spontaneous Control − Target Cell Spontaneous Control) / (Target Cell Maximum Control − Target Cell Spontaneous Control) × 100.

### NK cell cytotoxicity by colony formation assay

Cells (2,500 to 5,000) were plated, and on the next day NK cells were added at various ratios (1:1, 1:5, and 1:15, target cells: effector cells) (all samples in triplicates). After 4 hours of co-culture, NK cells were removed and fresh media was added into tumor cells. After 10 days of culture, colonies formed were visualized by crystal violet staining and were counted under microscope.

### PD-L1 measure by flow cytometry

A549P/A549CisR and H157P/H157CisR cells were stained with PE-PD-L1 Ab (BioLegend, 358103) (5 μl/10^6^ cells) (unstained cells as control) and the fluorescence was detected using the Canto II system (Becton-Dickinson, San Antonio, TX). In experiments with IL-6 addition, A549P and H157P cells were cultured and passaged in the presence of IL-6 (10 ng/ml), and P6 cells were used in analyses.

### Development of IL-6 knocked down and sc control cells by lentiviral transduction

For incorporation of siIL-6 RNA or scramble (sc) control plasmids into A549 and H157 cells, lentivirus construct carrying either sc or siIL-6RNA (pLenti-II vector, Applied Biological Materials Inc, Canada) was transfected into 293T cells with a mixture of pLent-II-siIL-6 RNA, psPAX2 (virus-packaging plasmid), and pMD_2_G (envelope plasmid) (4:3:2 ratio) using PolyFect Transfection reagent (Qiagen, 301305). After A549 and H157 cells were virally infected overnight, the culture media containing the virus was replaced with normal culture media, and maintained under normal cell culture conditions. After sub-culturing cells, the IL-6 knocked down cells were selected by culturing cells in the presence of puromycin (2 μg/ml) (Millipore, 540411) and then maintained in media containing 0.1 μg/ml puromycin.

### *In vitro* irradiation

For *in vitro* cell line irradiation, cells were plated 24 hours before irradiation and 6 Gy of irradiation was applied to the cells using a ^137^Cs source with a dose rate of 180–205 cGy/min. Zero to 24 hours after irradiation, cells were collected for migration/invasion assays or total RNA was extracted for qPCR analyses.

### *In vivo* xenograft studies

The luciferase tagged H157P and H157R24-1 cells (1 × 10^6^) that were obtained by transfection of luciferase reporter gene and selection procedure were orthotopically injected (1 × 10^6^ cells in media with Matrigel, 1:1 ratio in volume) into 8-week old female nude mice (NCI) (*n* = 6 per group). Tumor development and volumes were monitored once a week by *In Vivo* Imaging System (IVIS). Mice were divided into two groups. When luminescence reached to 5 × 10^5^ to 1 × 10^6^ radiance (p/sec/cm^2^/sr), mice of test group (lung site) were irradiated 5 Gy for 5 consecutive days [60] while remainder of the body was shielded from IR using lead blocks. Tumor size in irradiated group and control group were monitored twice a week by IVIS for 3 weeks. At the end of experiments, mice were sacrificed and tumors were obtained. All animal studies were performed under the supervision and guidelines of the University of Rochester Medical Center‘s Animal Care and Use Committee.

### Histology and immunohistochemistry (IHC)

Tissues obtained were fixed in 10% (v/v) formaldehyde in PBS, embedded in paraffin, and cut into 5-μm sections. Tumor tissue sections were deparaffinized in xylene solution, rehydrated, and immunostained with the IHC kit (Santa Cruz, sc2018). PD-L1 antibody was obtained from R&D (MAB1086). After staining, tissues were counterstained by Hematoxylin (Vector Laboratories, H3401). The average numbers of positively stained cells were obtained from careful counting of randomly chosen 3 different fields.

### RNA extraction and quantitative real-time PCR (qPCR) analysis

Total RNA (1 μg) was subjected to reverse transcription using Superscript III transcriptase (Invitrogen). qPCR was conducted using the appropriate primers and a Bio-Rad CFX96 system with SYBR green to determine the mRNA expression levels of genes of interest. Expression levels were normalized to GAPDH level.

### Western blot analysis

Cells were lysed in RIPA buffer (50 mM Tris-Cl at pH 7.5, 150 mM NaCl, 1% NP-40, 0.5% sodium deoxycholate, 1 mM EDTA, 1 μg/mL leupeptin, 1 μg/mL aprotinin, 0.2 mM PMSF). Proteins (20-40 μg) were separated on 8–10% SDS/PAGE gel and then transferred onto PVDF membranes (Millipore, Billerica, MA). After blocking procedure, membranes were incubated with primary antibodies (1:1000), HRP-conjugated secondary antibodies (1:5000), and visualized in Imager (Bio-Rad) using ECL system (Thermo Fisher Scientific, 34095). Antibodies used were; PD-L1 (R&D, MAB1086), p-JAK1 (pY1022, Assay Biotech, A7125), p-JAK2 (pY1007 + 1008, Abbomax, 601-670), p-MAPK (cell Signaling, 9101S), p-Erk (Cell Signaling, 4695), p-MEK (Ser 217/221, Cell Signaling, 9121), p-Akt (S473, Cell Signaling, 9271), and p-Stat3 (pY705, Abcam, ab76315), p-Stat1 (pY701, Abbomax, 620-160), p-Stat5 (Abcam, pY694, ab32364), p-NFκB (S536, Abcam ab86299), p-mTOR (Millipore, 09-345), JAK1 (Abgent, AP20699a), JAK2 (Abgent, AP20700c), Stat1 (Abgent, AP19835b), MEK (Ameritech, ATB-T2715), MAPK (Signalway, 21245), ERK (Ameritech, ATB-T5371), Akt (Santa Cruz, sc5298) and GAPDH (Cell Signaling, 2118S).

### Statistics

The data were presented as the mean± SEM. Differences in mean values between two groups were analyzed by two-tailed Student's *t* test. *p* ≤ 0.05 was considered statistically significant.

## SUPPLEMENTARY MATERIALS FIGURES



## Abbreviations

NSCLC: non-small cell lung cancer
NK: natural killer
PD-1: programmed death receptor-1
PD-L1: programmed death receptor ligand 1
APD-PD-L1: APC-conjugated PD-L1
NKG2D: NK group 2D
A549R26-1: A549 cells derived radioresistant
H157R24-1: H157 cells derived radioresistant
A549P: A549 parental
H157P: H157 parental
MAPK: mitogen activated protein kinase
MEK: mitogen-activated protein kinase kinase
Erk: extracellular signal-regulated kinase
mTOR: mammalian target of rapamycin
RT: radiotherapy
ULBP: UL16 binding protein
MICA: major histocompatibility complex class 1 chain molecule A
IHC: immunohistochemistry
TME: tumor microenvironment
LDH: lactate dehydrogenase
IVIS: in vitro imaging system.
