# Supplementary material for: Radiation alters PD-L1/NKG2D ligand levels in lung cancer cells and leads to immune escape from NK cell cytotoxicity via IL-6-MEK/Erk signaling pathway
Source: Oncotarget. 2017 Jul 12;8(46):80506–20. doi: 10.18632/oncotarget.19193 (PMC5655216; doi:10.18632/oncotarget.19193)
Supplement: Supplementary file 1 [file oncotarget-08-80506-s001.pdf]

Radiation alters PD-L1/NKG2D ligand levels in lung cancer cells and leads to immune escape from NK cell cytotoxicity via IL-6-MEK/Erk signaling pathway

SUPPLEMENTARY MATERIALS

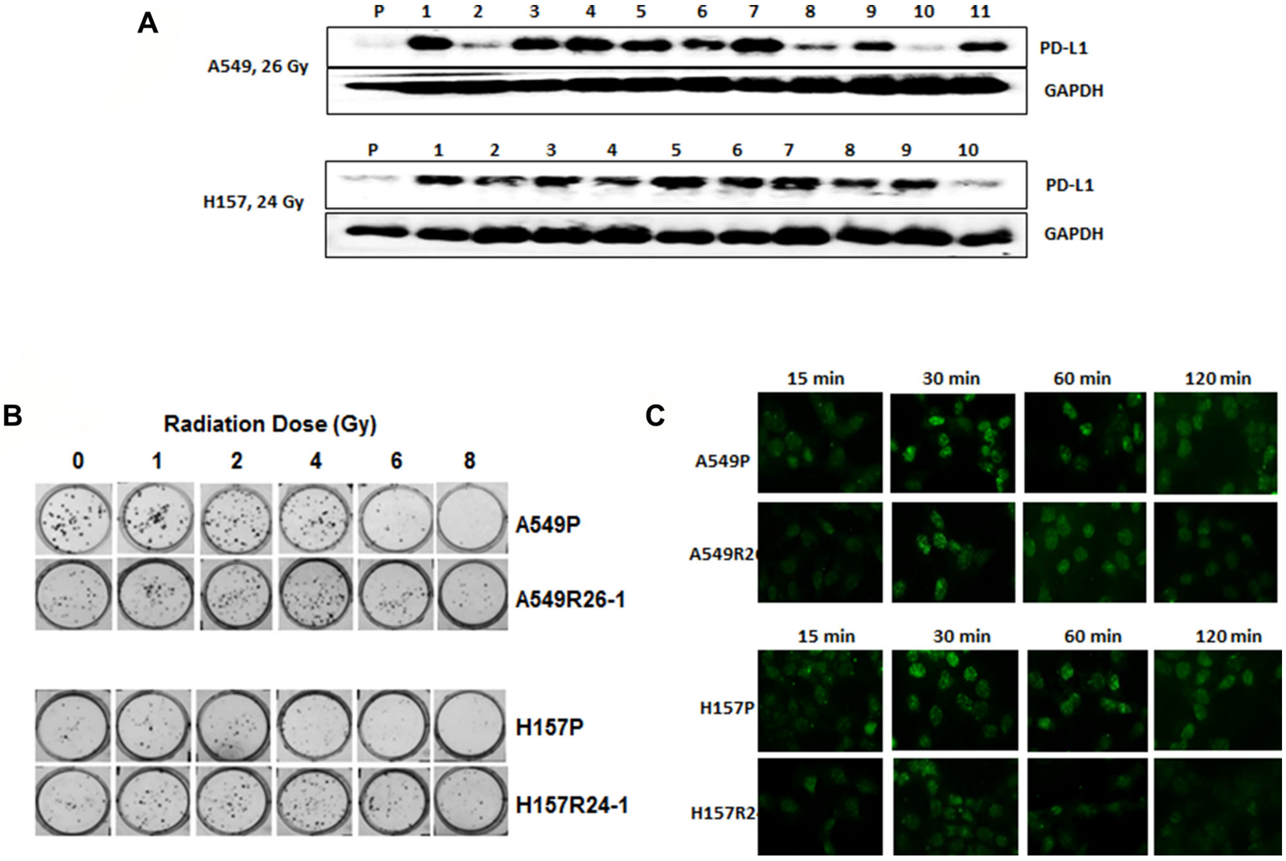

Supplementary Figure 1: Development of radioresistant sub-cell lines of NSCLC. (**A**) PD-L1 expressions in sub-line cells in cumulative 26 Gy-treated A549 and cumulative 24 Gy-treated H157 cells. (**B**) Images of 6-well plates used in clonogenic assays testing radioresistance of A549P vs. A549R26-1 and H157P vs. H157R24-1 cells. (**C**) Images of γH2AX staining of A549 vs. A549R26-1 and H157P vs. H157R24-1 cells at different time points after radiation (6 Gy).

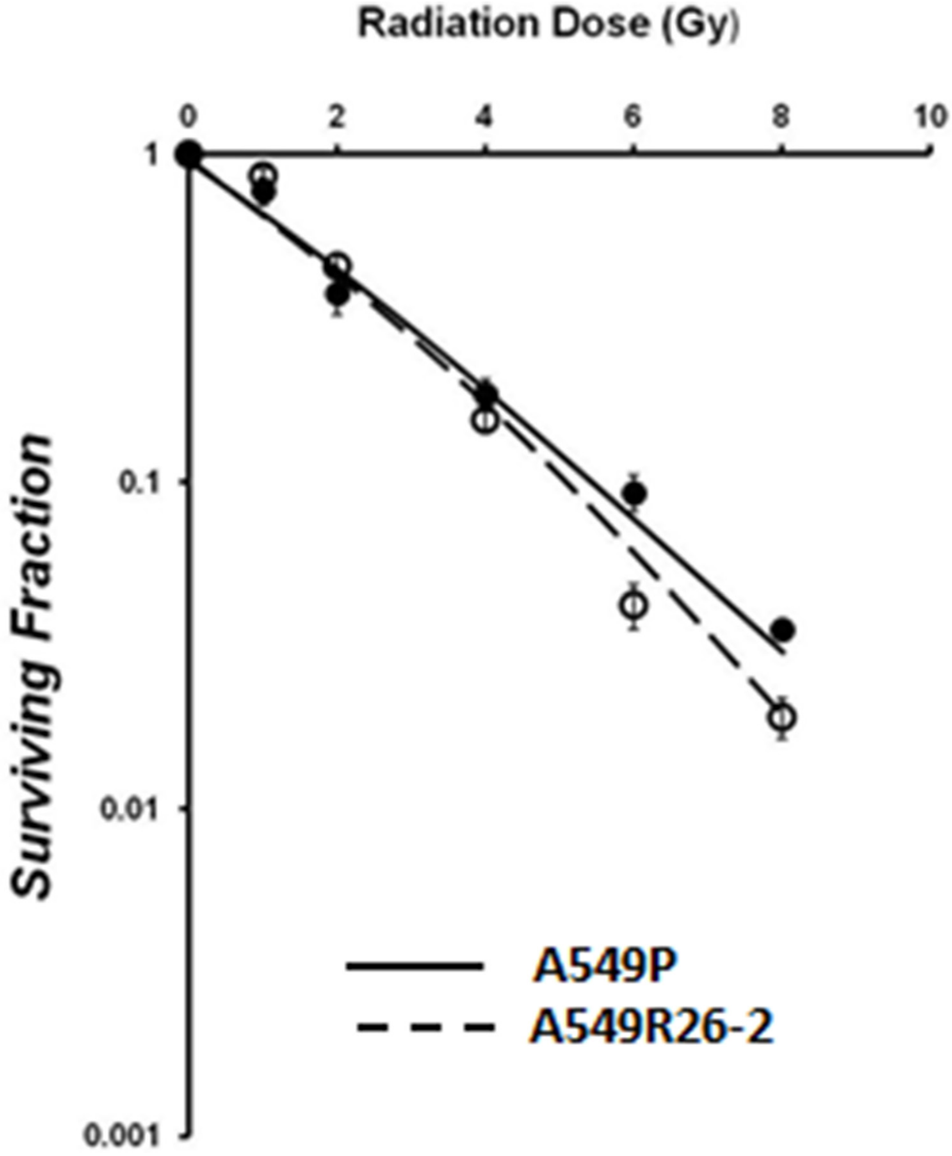

Supplementary Figure 2: Clonogenic assay of A549P vs. A549R26-2 cells.

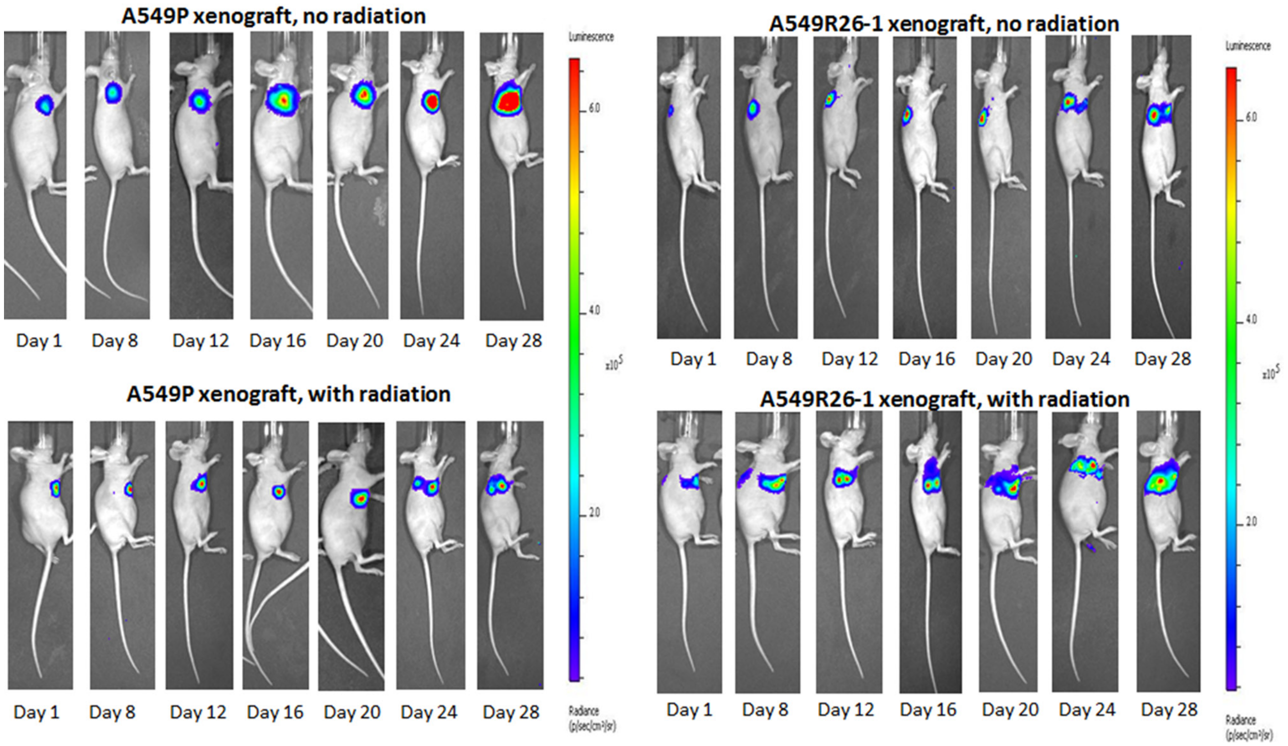

Supplementary Figure 3: IVIS Images of a representative mouse of each group showing tumor regression differences after radiation.
